# A High-Quality Mach-Zehnder Interferometer Fiber Sensor by Femtosecond Laser One-Step Processing

**DOI:** 10.3390/s110100054

**Published:** 2010-12-23

**Authors:** Longjiang Zhao, Lan Jiang, Sumei Wang, Hai Xiao, Yongfeng Lu, Hai-Lung Tsai

**Affiliations:** 1 School of Mechanical Engineering, Beijing Institute of Technology, 100081, China; E-Mails: zhaolongjiang@bit.edu.cn (L.Z.); wangsumei@bit.edu.cn (S.W.); 2 Department of Electrical and Computer Engineering, Missouri University of Science and Technology, Rolla, MO 65409, USA; E-Mail: xiaoha@mst.edu (H.X.); 3 Department of Electrical Engineering, University of Nebraska-Lincoln, Lincoln, NE 68588, USA; E-Mail: ylu2@unl.edu (Y.L.); 4 Department of Mechanical and Aerospace Engineering, Missouri University of Science and Technology, Rolla, MO 65409, USA; E-Mail: tsai@mst.edu (H.-L.T.)

**Keywords:** femtosecond laser, fiber sensors, Mach-Zehnder interferometer

## Abstract

During new fiber sensor development experiments, an easy-to-fabricate simple sensing structure with a trench and partially ablated fiber core is fabricated by using an 800 nm 35 fs 1 kHz laser. It is demonstrated that the structure forms a Mach-Zehnder interferometer (MZI) with the interference between the laser light passing through the air in the trench cavity and that in the remained fiber core. The fringe visibilities are all more than 25 dB. The transmission spectra vary with the femtosecond (fs) laser ablation scanning cycle. The free spectral range (FSR) decreases as the trench length increases. The MZI structure is of very high fabrication and sensing repeatability. The sensing mechanism is theoretically discussed, which is in agreement with experiments. The test sensitivity for acetone vapor is about 10^4^ nm/RIU, and the temperature sensitivity is 51.5 pm/°C at 200 ∼ 875 °C with a step of 25 °C.

## 1. Introduction

Modified fiber substrates are widely used in sensors [1–4] and wavelength filters [5–7] through coupling with planar waveguides or resonators. With its advantages of small size, light weight, electromagnetic interference immunity, wide bandwidth, and low transmission loss an optical fiber is a preferred platform for micro-sensors. Furthermore, fiber optics are widely used to interface various optoelectronic components. There are three main ways of transforming a single-mode fiber into a desired component: (1) grinding by using abrasive powders [8]; (2) processing by using a femtosecond (fs) laser [9–11], and (3) etching with HF solution. Many transparent materials irradiated by fs laser pulses are ablated with property changes due to the strong nonlinear ionization and corresponding free electron generation [12,13]. Some Fabry-Perot interferometers have been successively fabricated in optical fibers with fs lasers and used for refractive index sensing [9,11,14]. The fiber optic localized plasmon resonance (FO-LPR) sensor is also reported in which D-shaped fibers engraved by a fs laser is adhered with Au nanoparticles [10,15]. A multi-d-shaped optical fiber for refractive index sensing in a communication grade multimode optical fiber is fabricated by using a fs laser [16].

In this study, a fs laser was used to fabricate fiber sensors, producing a MZI structure on fibers with exciting potentials in high-quality sensing of refractivity-sensitive parameters such as temperature, concentration, humidity, pressure, stress and strain. Characteristic transmission spectra demonstrate that the fringe visibilities are all more than 25 dB. Fifty tested samples demonstrate the high fabrication and sensing repeatability of the simple sensing structure with some other advantages including reliability, compactness, robustness, high sensitivity, high flexibility, simple fabrication process and so on. The sensitivity for acetone vapor is about 10^4^ nm/RIU (refractive index unit), and the temperature sensitivity is 51.5 pm/°C at 200 ∼ 875 °C with a step of 25 °C.

## 2. Fiber Sensor Fabrication

**Figure 1.**
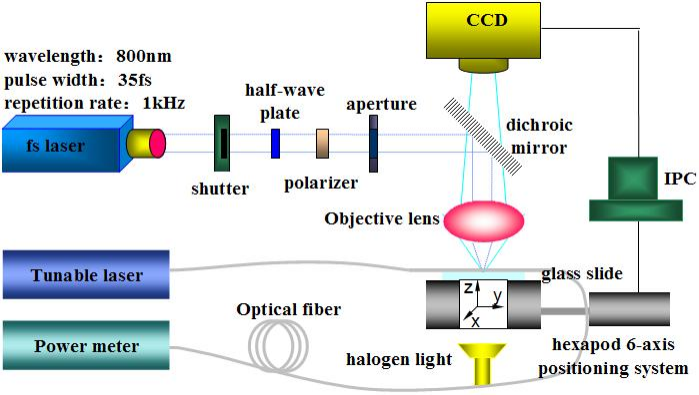
The scheme of the fs laser fabrication system.

The scheme of the fiber sensor fabrication system using a fs laser is shown in Figure 1. The central wavelength, pulse width and repetition rate of the fs laser (Spectra-Physics, Inc.) are 800 nm, 35 fs and 1 kHz, respectively.

**Figure 2.**
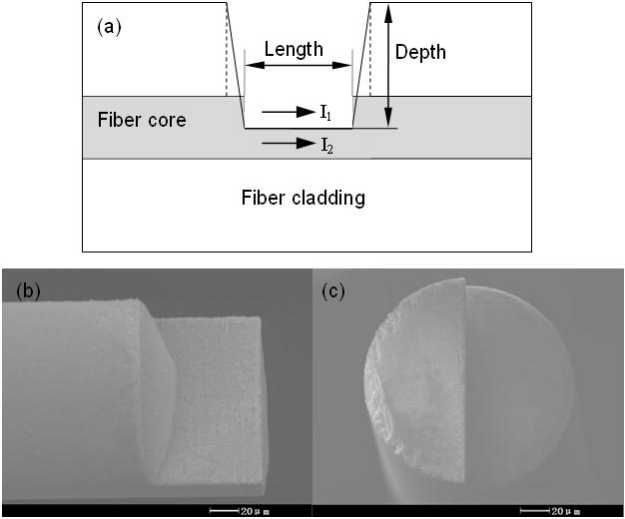
Trench fabricated by fs laser pulses. **(a)** Structural illustration. **(b)** Side view (a half part). **(c)** Cross section.

The laser pulse energy is attenuated through a half-wave plate and a polarizer to less than 50 μJ. Then, several neutral density filters are applied to reduce the pulse energy to less than 600 nJ before the objective lens. The attenuated fs laser beam is focused by an NA = 0.45 objective lens. The diameters of the single-mode fiber core and cladding are 8.2 μm and 125 μm respectively. The effective refractive index of the fundamental mode at 1,550 nm is 1.4682, and the calculated physical refractive indices of the fiber core and the cladding are about 1.4712 and 1.4659 respectively. A detection system (Agilent 8163B) consisting of a tunable laser and an optical power meter is employed to monitor the transmission spectra by wavelength sweeping. Figure 2 shows the side-ablated structure (an in-line trench) on the single-mode fiber with a length of about 75 μm. During by fs laser processing of fibers, nitrogen gas is used to blow off debris.

**Figure 3.**
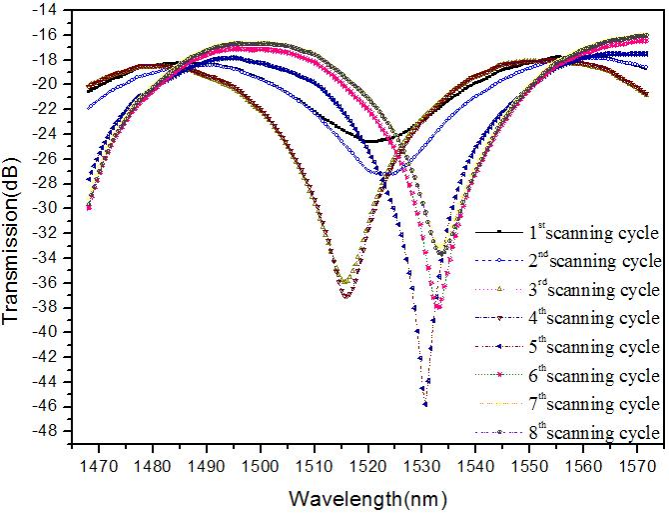
Transmission spectra evolution of the sensor structure during the 1^st^–8^th^ scanning cycle.

The transmission spectra of the side-ablated fiber were real-time tested during the fs laser fabrication process. The tunable laser continuously scans through its wavelength range (1,465–1,575 nm) at the rate of 0.5 nm per step. In each scanning cycle of fs laser ablation, a layer in x-y plane (as shown in Figure 1) is exposed to laser irradiation. Transmission spectra evolutions of structures from the 1^st^ scanning cycle to the 8^th^ scanning cycle are shown in Figure 3. The transmission spectra of the processed fiber keep changing in each processing cycle by fs laser ablation. In the 1^st^ scanning cycle, the ablation depth is about 60 μm, which results in an attenuation band with a relatively low loss. The processing cycle is repeated eight times at the same depth. The losses of the attenuation bands increase from the 1^st^ scanning cycle to the maximum at the 5^th^ scanning cycle and then decrease from the 6^th^ to the 8^th^ cycle. The interference dip wavelength shifts during the ablation processes while FSR nearly keeps constant. From the 1^st^ cycle to the 8^th^ cycle, debris decreases gradually. Fifty fabricated samples demonstrate high repeatability of the trench structure and its sensing properties.

## 3. Results and Discussion

**Figure 4.**
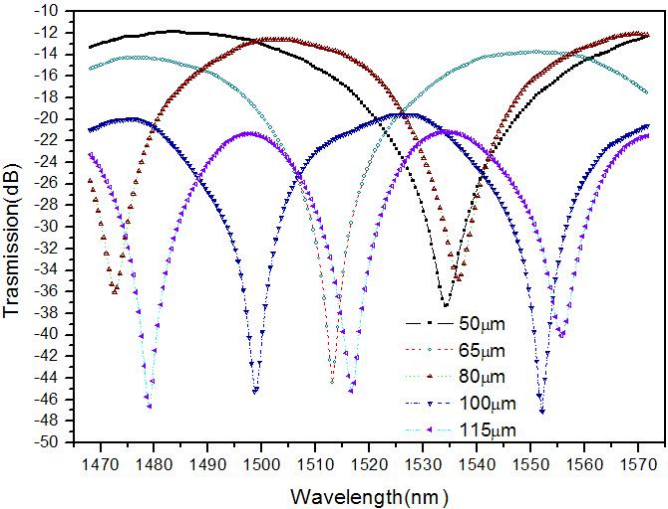
Transmission spectra of the structures at different lengths.

Fifty MZI fiber sensors are fabricated with the same trench depth of 60 μm but different lengths of 50, 65, 80, 100 and 115 μm. As shown in Figure 4, the fringe visibilities of the processed fibers are all greater than 25 dB. The background loss increases as the trench length increases and it is greater than 11 dB in all the cases, which is similar to the previously reported experiment [1]. The relatively high loss may be mainly due to the light scattering at the laser-ablated surface [11]. At the trench lengths of 50, 65, 80, 100 and 115 μm, the FSRs are about 101, 74, 64, 53.5 and 38 nm, respectively. This indicates that FSR decreases as the trench length increases, which implies more interference orders at longer trench lengths.

The fabricated structure forms a MZI whose two main light transmission paths are (1) the remaining D-type fiber core; and (2) the cavity in the trench. The interference intensity is expressed by [17]:

(1) $$I=I_{1}+I_{2}+2\sqrt{I_{1}I_{2}}\text{cos}\varphi$$

where *I*_1_ and *I*_2_ are the intensities along the two light paths and *ϕ* (= 2*π*Δ*n_eff_ L*/*λ* + *φ_0_*) is the phase difference; Δ*n_eff_* (≈ 0.4682) is the difference between effective refractive index of the D-type fiber core and that of the trench cavity; *λ* is the wavelength; *L* is the trench length; and *φ_0_* is the initial interference phase. The fringe visibility depends on *I*_1_ and *I*_2_, and is optimized when *I*_1_ = *I*_2_. The interference changes in each ablation scanning cycle are shown in Figure 3.

According to Equation (1), the phase difference of two adjacent minimum interference signals is 2π. Therefore:

(2) $$\left(2\pi \Delta n_{\mathrm{eff}}L/\lambda_{1}+\phi_{0}\right)-(2\pi \Delta n_{\mathrm{eff}}L/\lambda_{2}+\phi_{0})=2\pi$$

where λ_1_ and λ_2_ are the wavelengths corresponding to the two adjacent minimum interference signals.

Thus, the trench length is:

(3) $$L=\lambda_{1}\lambda_{2}/\left(\Delta n_{\mathrm{eff}}\left(\lambda_{2}–\lambda_{1}\right)\right)$$

which shows that the FSR decreases as the trench length increases. Based on the interference spectra in Figure 4, the calculated trench lengths are 46.5, 62.8, 75.5, 92.8 and 126.1 μm, which are reasonably close to the experimental results: 50, 65, 80, 100 and 115 μm, respectively. The errors may mainly be caused by the simplification that the cladding effects and variation of Δ*n_eff_* are not considered.

**Figure 5.**
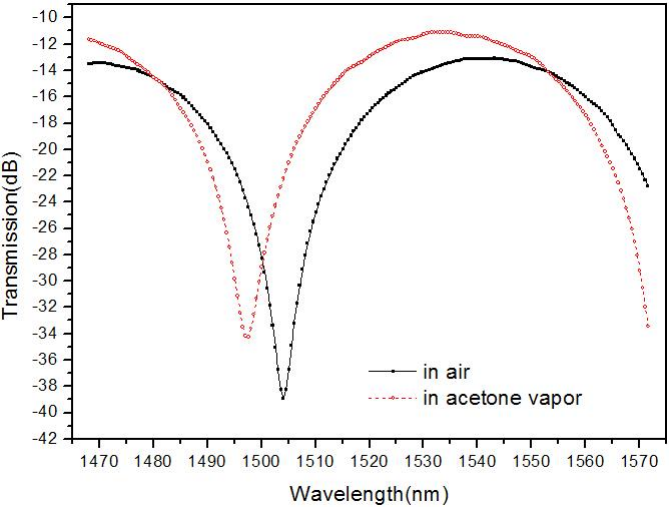
Sensing test results in air and acetone vapor at room temperatures.

Gas sensing tests in air and acetone vapor were conducted. The sensor with a trench length of about 80 μm was put into a sealed stainless steel tube. The inner diameter and the length of the stainless steel are about 1 cm and 20 cm, respectively. The sensor transmission spectrum in air at room temperature is shown in Figure 5.

Then, 1.5 mL acetone was injected into the stainless tube. The transmission spectrum of the sensor in the acetone vapor was measured at room temperature. The spectrum scanning procedure is repeated several times until there is no obvious change compared with the preceding ones and the final sensor spectrum in acetone vapor is also shown in Figure 5. The refractive index of acetone vapor is greater than that of air, between which the difference is on the order of magnitude of 10^−4^ RIU. Compared with the results in air, the interference dip wavelength shift in acetone vapor is about 6.5 nm. The sensitivity is about 10^4^ nm/RIU for acetone vapor.

**Figure 6.**
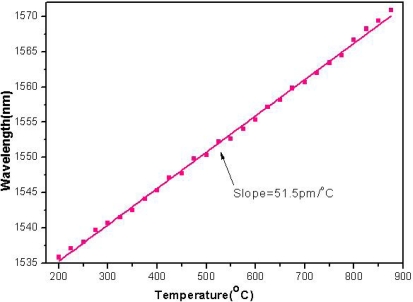
The temperature sensing property of the sensor.

Temperature measurements were also conducted by using the proposed fiber sensor. The sensor with a trench length of about 85 μm was selected. The temperature changes from 200 °C to 875 °C at a step of 25 °C. The interference dip wavelength shows a red shift with the increase of the temperature, as shown in Figure 6. It is mainly due to the change of Δ*n_eff_* or the effective refractive index change of the D-type fiber caused by the temperature variation. The temperature sensitivity estimated by least square linear fitting is 51.5 pm/°C.

## 4. Conclusions

This paper reports a MZI sensor ablated by fs laser pulses in single-mode fibers, which demonstrates exciting high-quality for both temperature and chemical sensing. For 50 processed samples, the sensing structure showed very high fabrication and sensing repeatability, and structures with the desired proprieties are successfully obtained in every experiment. The sensing mechanism is discussed theoretically, and is in agreement with the experimental results. The fringe visibility of the structure is more than 25 dB. The sensitivity for acetone vapor sensing is about 10^4^ nm/RIU. The sensor was applied for high temperature measurements with a sensitivity of 51.5 pm/°C at 200 ∼ 875 °C.
